# Statistics teaching in medical school: Opinions of practising doctors

**DOI:** 10.1186/1472-6920-10-75

**Published:** 2010-11-04

**Authors:** Susan Miles, Gill M Price, Louise Swift, Lee Shepstone, Sam J Leinster

**Affiliations:** 1 School of Medicine, Health Policy and Practice, Faculty of Health, University of East Anglia, Norwich, NR4 7TJ, UK

## Abstract

**Background:**

The General Medical Council expects UK medical graduates to gain some statistical knowledge during their undergraduate education; but provides no specific guidance as to amount, content or teaching method. Published work on statistics teaching for medical undergraduates has been dominated by medical statisticians, with little input from the doctors who will actually be using this knowledge and these skills after graduation. Furthermore, doctor's statistical training needs may have changed due to advances in information technology and the increasing importance of evidence-based medicine. Thus there exists a need to investigate the views of practising medical doctors as to the statistical training required for undergraduate medical students, based on their own use of these skills in daily practice.

**Methods:**

A questionnaire was designed to investigate doctors' views about undergraduate training in statistics and the need for these skills in daily practice, with a view to informing future teaching. The questionnaire was emailed to all clinicians with a link to the University of East Anglia Medical School. Open ended questions were included to elicit doctors' opinions about both their own undergraduate training in statistics and recommendations for the training of current medical students. Content analysis was performed by two of the authors to systematically categorise and describe all the responses provided by participants.

**Results:**

130 doctors responded, including both hospital consultants and general practitioners. The findings indicated that most had not recognised the value of their undergraduate teaching in statistics and probability at the time, but had subsequently found the skills relevant to their career. Suggestions for improving undergraduate teaching in these areas included referring to actual research and ensuring relevance to, and integration with, clinical practice.

**Conclusions:**

Grounding the teaching of statistics in the context of real research studies and including examples of typical clinical work may better prepare medical students for their subsequent career.

## Background

The UK General Medical Council (GMC) sets out, and periodically revises, the standards which undergraduate medical students must obtain by graduation. Whilst not providing specific directions as to the level, amount or content of statistics teaching, the 2003 'Tomorrow's Doctors' document (which medical schools are working under at the time of writing) clearly states that graduates need to be able to "integrate and critically evaluate (scientific) evidence", "know about biological variation, and have an understanding of scientific methods, including both the technical and ethical principles used when designing experiments", be able to "evaluate effectiveness (of treatment) against evidence", "analyse and use numerical data", and "use research skills to develop a greater understanding and to influence their practice" (paragraphs 13,15,16,26) [1]. The 2009 document is no more specific about what medical students need to learn about statistics and research methods, referring to a need to "Apply to medical practice the principles, method and knowledge of population health and the improvement of health and healthcare" (section 11), and "Apply scientific method and approaches to medical research" (section 12). This includes being able to "critically appraise ... studies reported in the literature", "formulate simple research questions ... and design appropriate studies", and "apply findings from the literature to answer questions raised by specific clinical problems" [2].

Published accounts by those involved in teaching medical statistics [3,4] support anecdotal experience that statistics is not the most well-liked subject in the undergraduate medical curriculum and that, despite the GMC guidance, some students tend not to perceive these subjects as relevant to medical practice. In 1987, the GMC identified failure to recognise the relevance of teaching at the time as one of the barriers faced when teaching community medicine, including medical statistics [5].

Several papers describe the history of statistics teaching to medical students in the UK [3,6,7]. In their 1957 recommendations for medical education, the GMC noted only the desirability that students "be acquainted with the principles governing the design and interpretation of clinical trials" [8]. By 1967 they were explicitly stating a need for instruction in "biometric methods" and data analysis [9]. This suggests that doctors would be expected to perform statistical analyses themselves.

In 1987, the findings of a GMC Education Committee Working Party indicated that current GMC recommendations were not being well implemented by medical schools in several areas, including the teaching of medical statistics [5]. Thus, during the 1990s discussion amongst medical statisticians concerning teaching statistics to undergraduate medics no longer focussed on whether it needed to be taught, but on what and how medical statistics should be taught [10-12]. Altman and Bland [3] suggested that whilst reading and interpreting research is the main reason that doctors need to know about statistics, the everyday need to absorb pharmaceutical company literature and understand diagnostic test results requires statistical understanding. The advent of Evidence Based Medicine (EBM), supported by easier internet access to research literature for both doctors and patients, brought an increased need for critical appraisal. EBM added momentum to the changing aims of statistics teaching [7], with an increased emphasis on concepts and a move away from techniques [6], and raised the question of whether teaching should be directed towards "consumers of research" or "producers of research" [13]. Despite the increasing focus on teaching statistics there is evidence of continuing misunderstanding of basic statistical concepts among practising clinicians and medical researchers [3].

Almost all discussion on this issue has been conducted by medical statisticians with little involvement from recipients of statistical training, the doctors themselves. This study aimed to elicit the opinions of practising doctors on why, what, how and when statistics should be taught to medical undergraduates. Ultimately this research aimed to inform educators implementing GMC guidance as to how undergraduate teaching of probability and statistics can best prepare medical students for their role as doctors.

## Methods

### Questionnaire

A questionnaire was designed to investigate doctors' use of, and attitude, to statistics and probability (full questionnaire available from corresponding author). The specific questions included aimed to gather information about the doctors' experiences with probability and statistics teaching during their undergraduate training, their use of probability and statistics in their daily working life, and their views (based on both of these sets of experiences) on what and how statistics and probability should be taught to current undergraduate medical students. The questionnaire comprised 5 sections. In Part A participants were asked to indicate their current position, provide the year they achieved their undergraduate medical qualification, details of any other post-graduate qualifications achieved, and indicate the level of any involvement in health research. Part B concerned doctors' use of probability and statistics in their current practice and their attitudes towards probability and statistics, and towards maths at school, The results of Parts A & B are reported separately [14]. In this article we focus on the questions which asked doctors about their own undergraduate teaching (Part C) and what undergraduate teaching they thought current undergraduates should receive (Part D) (Table 1). Part E was an optional test of statistical knowledge, on which the participants could receive feedback if they wished by providing their email address; the results of Part E have not been reported. Development of the questionnaire included all five authors (which includes three statisticians with experience of teaching, including teaching statistics to undergraduate medical students, one expert in medical education and one attitude researcher) and a colleague (with expertise in research methods and systematic reviewing). The questionnaire was piloted with clinical colleagues at the University of East Anglia (UEA) Medical School and outside the university setting. Volunteers were asked to provide any comments they had about the questionnaire, including clarity of questions, length of the questionnaire and adequacy of response options. The questionnaire was set up as a form using Microsoft Word, so it could be emailed as an attachment to potential participants.

**Table 1:** Questions reported in this article *(response options)*.

C1 "Were probability and statistics taught on your undergraduate course?" (Yes, No, I can't remember)
C5 "Did the teaching seem useful at the time?" (Yes, No, I can't remember)
C6 "Has the teaching been relevant to your subsequent career?" (Yes, No, I don't know)
C7 "Please suggest any ways in which your undergraduate teaching in probability and statistics could have been more useful" (open ended)
D1 "What teaching do you think that current undergraduate medical students should receive in probability, statistics, epidemiological methods and related numerical methods and skills?" (open ended)

Full questionnaire available from corresponding author

### Procedure

The questionnaire and participant information sheet were sent by email in April 2007 to doctors on a distribution list of 'Recognised Teachers' at UEA Medical School, UK. The distributions list comprises UEA MB/BS faculty, medical doctors, allied health professionals and experts in various areas e.g. medical lawyers, who contribute to both placement- and university-based teaching. Specifically, the doctors on this list are clinicians from general practices and hospitals who are involved in teaching students on placement at their institutions. The original list (n = 682) was edited to exclude anyone obviously not a doctor, based on job title and personal knowledge of the research team. However, it was extremely likely that the final distribution list (n = 473. 33% general practitioners, 54% consultants, 13% other medically qualified or unknown) still included non-medics, thus only those with medical qualifications were asked to respond. Two email reminders were sent before the deadline of three weeks. Consent was assumed to have been given on return of a completed questionnaire. The study was approved by the UEA Faculty of Health Research Ethics Committee.

### Analysis

Content analysis of the qualitative data was conducted by two of the authors to ensure robust coding. Responses were grouped initially by one author (SM) into closed categories emergent from the data. These categories comprised single words or phrases that described and summarised all the responses given. Any participant's response could appear under several categories if mentioning several ideas relevant to different category themes; thus, there were more comments than participants. This initial categorisation was followed by merging or splitting of categories and checking appropriateness of coding. After this, a second coder (GMP) checked the coding. Finally, differences in ideas about coding, including naming and constitution of categories and classification of responses were discussed until both coders agreed. A list of recommendations for the teaching of statistics to medical students was developed through discussion among the authors of the final list of category themes.

## Results

### Characteristics of participants

130 doctors responded by the deadline (27% response rate); of these 79 (61%) were consultants in various specialities, 33 (25%) were general practitioners, and 18 (14%) were a mix including junior doctors, specialist registrars and clinical academics. Their qualifying year ranged from 1951-2004; 67 (52%) qualified in or after 1984 and 13 (10%) in or after 1994. Forty-six (35%) participants had a post-graduate research qualification (MD, MPhil or PhD). Seventy-three (56%) of the participants had analysed numerical data from a research study, 13 (10%) had never been involved in any health research, and the remainder (44, 34%) had either been involved in the design and planning of a study, recruiting or treating patients on a study, and/or conducted their own undergraduate student research, but had not analysed data in any case.

### Participants' experience of undergraduate teaching

Eighty-two (63%) participants remembered being taught probability and statistics as an undergraduate medical student. However, of these 82 participants, only 33 (40%) remembered it as seeming useful then; 38 (46%) said it had *not *seemed useful at that time. In contrast, 60 of these 82 participants (73%) felt this teaching had in fact been relevant to their subsequent career.

### Improving undergraduate teaching for the future

When asked to suggest ways in which their undergraduate teaching in probability and statistics could have been more useful (Question C7), 68 participants (52%) provided suggestions. Two major themes emerged: (i) that teaching could have been made more relevant to future practice (n = 49) and (ii) that the style and format of teaching could have been different (n = 26).

When asked what teaching they thought current undergraduate medical students should receive (Question D1) 114 participants (88%) provided a response. The same two major categories appeared as for C7: namely (i) that the teaching needs to be clearly relevant and applied to future practice (n = 37), and (ii) suggestions as to how teaching should be organised and run (n = 17). Responses to D1 also gave rise to a third major category addressing the curricular content that participants thought should be covered in future teaching (n = 81), which was not present in responses to C7. Due to the similarities in responses to both of these questions, discussion of the results addresses both questions together. Quotes are in Tables 2, 3 and 4; question number and participant's study ID in parenthesis.

**Table 2:** Make teaching relevant to future practice

*General*
"Making it seem more relevant - it seemed really pointless at the time" (C7:241)
"If the need for this teaching in a doctor's professional career had been made more obvious" (C7:384)

*Clinical practice*
"Discussion of uses in day-to-day clinical work. Methods of explaining probabilities to patients and students" (C7:91)
"In my course not particularly linked at that time to clinical scenarios ... Explicit working through of cases and the decisions based on evidence available and risks and benefits I think would have been useful" (C7:190)
"They should learn enough to understand the validity, importance and relevance of research into all aspects of clinical policy and practice: aetiology, diagnosis, therapy, prognosis, as well as understanding research synthesis and guidelines. This should be integrated with clinical care and reflective practice. ... For example, students studying the nervous symptom [system] may be invited to look at studies of diagnostic accuracy for MRIs in multiple sclerosis and to look at RCTs for pharmacological interventions. Learning to take into consideration the sensitivity and specificity of clinical signs should be as important as knowing how to examine for them." (D1:200)

*Research*
"Forget the mathematics concentrate more on interpretation of papers" (C7:188)
"If it had been related to real research" (C7:404)
"Being made topical i.e. relating to current trials" (C7:341)
"Applied methods relevant to interpreting research papers and pharmaceutical company material" (D1:267)
"They need a thorough grounding in these subjects so that they can read papers critically and do audit and/or research with confidence" (D1:171)

**Table 3:** Teaching style, format and organisation

"If we were taught practical applications of these things at the outset and expected to apply the knowledge throughout the training period." (C7:71)
"More practise and seeing senior colleagues using them in practice" (C7:161)
"More time needed. Lectures were regarded as difficult to understand by most of my peers." (C7:209)
"Rather than just formal lectures during a set 2 weeks, medical statistics needs to be fully integrated into all parts of the curriculum so that research methods and results can be understood in all aspects of clinical medicine" (C7:455)
"More problem solving type work, small group or 1-1 even" (C7:15)
"Devote time to teaching the subject and start simply and gradually build up towards the final year."(D1:455)
"Very practical problem based teaching, including general practice/secondary care" (D1:183)

**Table 4:** Curricular content

*Basic grounding*
"Understanding of basic concepts and data interpretation" (D1:144)
"A basic grounding - population distributions, understanding p-values, confidence intervals, concepts of risk" (D1:15)
"Teaching in the core principles and its application and relevance in current clinical practice" (D1:238)
"They need comprehensive teaching in understanding statistics, epidemiology and critical appraisal as part of EBM. They do not need to learn how to calculate things that can come later" (D1:49)

*Specific methods*
"Basic probability and statistics up to an understanding of regression (not doing!), basic research methods with understanding of different epidemiological designs ... basic analysis of data using a statistical software package" (D1:194)
"I think all doctors should be able to recognise the basic statistical errors which crop up all the time in research - e.g. using correlation as an indicator of causality. All need to be able to describe relative risks e.g. to know what is the risk of everyday activities (driving etc) so that this can be compared to a risk of treatment or disease usefully to a patient. ..." (D1:260)
"As a minimum undergraduates should know why statistical methods are important, how to describe probability and the basis of simple tests such as t tests. They should know what confidence intervals mean." (D1:118)
"Should be able to understand probability, standard deviation and tests for statistical significance" (D1:449)
"Types of study with relevance to the question; levels of evidence; understanding of meta-analyses; simple stats. e.g. p, CI, parametric/non parametric; common tests; 4 × 4 tables, sensitivity/specificity" (D1:131)
"As now but more on 1) theory of probability and risk, 2) multiple regression and 3) diagnostic inference" (D1:18)

### (i) Make teaching relevant to future practice (Table 2)

In response to question C7, some participants (n = 18) simply stated that the teaching they received would have been improved if it had been more relevant to future practice, but without further details. It was evident from their responses that some of these participants had not recognised the value of this teaching at the time. Other responses indicated that teaching should be made relevant under two key areas of application: clinical practice and research.

The first key area of teaching relevance was future day-to-day working clinical practice, including clinical decision-making and explaining risk to patients (C7: n = 18; D1: n = 16). The second key area of application was to real research studies. Teaching should enable students to critically review published research, data from clinical trials and information about new treatments and drug literature (C7: n = 17; D1: n = 24). Answering Question D1, 6 of these participants specifically commented that teaching should enable students to conduct research and audit themselves.

### (ii)Teaching style, format and organisation (Table 3)

Comments categorised as relating to pedagogy tended to be specific to the participant's own experience and consequently more diverse and difficult to interpret. Suggestions included: starting the teaching early in the course, and building on this throughout the whole course (as opposed to a block of teaching in one year); having enough teaching (more than participants had experienced themselves); pitching it at an understandable level (making allowances for different ability levels); integrating with other subject areas including clinical; making it practical and interactive; and teaching methods including lectures, seminars, problem-based learning (PBL).

### (iii) Curricular content (Table 4) [in response to D1 only]

Forty-three participants noted that current teaching should give students a basic grounding or understanding. Of these, 24 provided no further details; but 19 additionally suggested that this basic grounding should be applied/made relevant to future practice, and/or referred to specific topics that they felt should be covered.

Twenty-four participants outlined details of *topics *that they thought students should learn. These included mention of epidemiology (n = 6); probability, uncertainty and risk (n = 15); descriptive statistics including variability (n = 4); statistical significance including confidence intervals (n = 7); statistical tests and measures of effect (n = 12). Whilst a few participants did identify particular data analysis techniques that they felt current undergraduate students should be taught, the methods mentioned were varied and tended to be specific to the needs of the participant in question.

## Discussion

Our findings suggest that, whilst less than half of doctors recognised the value of their own undergraduate training in probability and statistics at the time (40%), the majority (73%) had found their learning relevant to their subsequent career, in support of GMC recommendations [1,2].

Participants offered informative suggestions both as to how their own undergraduate training could have been improved, and for the content and structure of future teaching. Responses were dominated by a desire that statistical topics be more applied to clinical work and to interpreting research. The separation of application of statistics to clinical and research demonstrates progressive departure from a historical view [3] of the need for medical students to learn about statistics only in case they conduct research during their medical career. This latter approach has tended to alienate students from the topic because they did not envisage becoming involved in research, thus they did not see any need to analyse data themselves (contrary to GMC guidelines) except perhaps in a student-selected study module if required. The quote from one participant "*Any measures that link the statistics to clinical work rather [than] research work will help*" (D1:3) reflects remnants of this historical legacy. Teachers of medical statistics have accordingly recommended focusing on interpretation and understanding of concepts while keeping mathematical formulae and calculation to a minimum [3,4,6,7,11,13].

Increasingly, however, the boundaries between clinical and research applications of statistics in clinicians' working lives have become blurred since all clinicians are now urged to practise medicine in an evidence-based way, and EBM embeds interpretation ('consumption') of medical research (requiring an understanding of statistical concepts) into clinical decision-making. Another participant's comment reflects this: "*Working as a clinician, irrespective of research activity, is difficult without any working knowledge of statistics*" (D1:340). Teaching needs to ensure that medical students appreciate the relevance of learning the skills required for critical appraisal of medical research literature. Referring to a UEA medical student, one participant commented: "*One of my personal advisees ... was astonished that we actually used critical appraisal skills in our day to day work - she thought that this was a hurdle to get over in med school followed by a blissful career free of any appraisal of literature.*" (D1:375). As previously reported [14] today's doctors use statistics and probability for a wide range of activities, including: explaining levels of risk to patients, accessing clinical guidelines and evidence summaries, assessing medical marketing and advertising material, interpreting screening test results, reading research publications for general professional interest, using research publications to explore non-standard treatment and management options, and for analysing numerical data.

One of the recommendations by the practising doctors is to assert the relevance of statistical and research methods, as well as designing curricular content and methods of delivery to illustrate this. The need to demonstrate relevance by educators is perhaps typical of teaching 'service' subjects in applied or vocational courses. The introduction of PBL in medical schools provides an opportunity for at least some statistics learning to be integrated within other parts of the course to help students perceive the subject as relevant to clinical practice and playing a vital part in the evidence base. Whilst acknowledging the practical difficulties associated with truly integrated teaching, Bland [15] argues that failure to incorporate statistics and research methods into the central curriculum will lead to marginalisation. He notes that some statistical concepts could be included in patient-based cases (e.g. intervals for diagnostic accuracy), research papers could trigger PBL questions about the condition as well as the research methodology used, as could news articles presented by the patient. Such integration would operate alongside conventional teaching methods, such as seminar-based question and answer sessions with a medical statistician.

Recommendations to introduce the concepts early in the medical course, then using and reinforcing throughout the course is in-line with findings that early one-off training is associated with later poor knowledge [16]. If students encounter the topics and learn skills in their first or second year but not in the rest of their undergraduate training it is not surprising that they lose the skills and also any impression of relevance.

Suggestions by the participating doctors indicated that a basic level of fluency with probability and risk, descriptive statistics, measures of diagnostic accuracy, application and interpretation of elementary hypothesis tests, epidemiological concepts and study design would be a useful foundation for undergraduates to build on later in their careers. If the aim is for graduates to be able to do research or analysis themselves then the approach to course content probably needs to be broader and deeper. However, only a few doctors in our survey specifically mentioned the need to be able to do research or data analysis as a learning goal; this is not in line with the 2003 GMC [1] recommendation that students should be able to 'analyse and use numerical data'. Student research projects provide an opportunity for students to develop their grasp of statistics from interpretation (the aim implied or stated by most respondents) into carrying out. But whether all students need to be taught details of all statistical tests is questionable, and is not evident in the more recent GMC recommendations [2]. An on-demand system with advice from medical statisticians may be more appropriate to enable students to adequately conduct statistical analysis for the needs of their particular research project. It is critical to remember that perceived need plays a strong role in students' receptiveness to acquiring these skills.

It is important to see the doctors' suggestions in context; 90% of the sample graduated before 1994 so the statistics teaching they received may have been more limited and with a different conceptual basis than that of today's students and more recent graduates. However, regardless of when they trained, what training they received at that time and any perceived deficiencies in that training, the participants' responses to question D1 ("What teaching do you think that current undergraduate medical students should receive in probability, statistics, epidemiological methods and related numerical methods and skills?") will likely have been driven by their current requirements as practising doctors, and consequently these responses provide valuable information about the knowledge and skills graduates need from a valid perspective. Seniority may make them less research- and technology- aware than the general population of doctors. On the other hand their university teaching connection may be associated with being more research-active, which might bias their opinions in favour of the benefits of statistical skills, in comparison to the 'average' doctor. Their experience of teaching medical students should, however, make their suggestions for undergraduate statistics teaching all the more valid. One limitation of this study is the likelihood of recall bias implicit in asking doctors to remember undergraduate teaching that may have taken place a considerable number of years ago. In particular, this would impact on recollection of the timing and amount of undergraduate teaching, and differentially so for those who may have taken an interest in the subject at that stage or later. It may also mean that participants cannot precisely remember when they were taught a particular statistical skill, i.e. at undergraduate or postgraduate level. However, the questions included in this study were not limited to recall of past teaching, but included opinions about current teaching informed by their experience of practice until the present. Future research could usefully examine the views of practising doctors closer to their undergraduate training (e.g. Year 2 Foundation programme doctors) and doctors with less teaching and/or research experience. The percentages of consultants and GPs in the original invited list (33% and 54% respectively) are broadly in line with the corresponding percentages (25% and 61%) of the responding sample. Formal comparisons between the responses of the consultants and GPs conducted for other parts of the questionnaire in the previously published article did indicate some differences in the frequency at which they did activities that might require the use or knowledge of probability and statistics, and in how useful they judged probability and statistics in their own work [14], However, examination of the qualitative data reported here indicated no differences between the consultants and GPs in the information they provided about how their own teaching could have been more useful or the recommendations they provided for current undergraduate teaching.

Whilst acknowledging the limitations of the small and local sample, with its undergraduate teaching associations, the findings of this research are supported by the literature, as detailed above. Thus, it is possible to make some recommendations for future undergraduate statistics teaching in medical schools that can be used to improve existing teaching programmes.

## Recommendations for statistics teaching in medical school

The findings of this study taken on their own do not provide sufficient evidence to inform the development of a detailed syllabus for teaching statistics to medical undergraduates. However, they do suggest certain principles that should be applied in the development, or redevelopment, of an effective curriculum.

• Use findings, such as those reported in this paper and in Swift *et al*. [14], to demonstrate to students that practising doctors believe that learning about probability and statistics, and associated research design and analysis skills is relevant to their actual daily work as a doctor; and not just for those doctors who intend to conduct their own research.

• Highlight the wide variety of areas in which statistics and related knowledge/skills is required and beneficial for the practising doctor.

• Use examples of how statistics and related knowledge/skills is used in daily clinical practice: e.g. explaining risk to patients, comparing potential treatments, interpreting diagnostic test results, interacting with drug reps and reading pharmaceutical literature for new medications, and understanding material brought in by patients about treatments they are interested in pursuing. Involving clinicians in the identification of suitable material and/or teaching may be beneficial to further demonstrate to students the relevance of statistics to the average doctor. For instance, tutors could use medical technology or pharmaceutical advertising material as basis to discuss with students the implications of a claim that a product is 'clinically proven' to be effective. The original published data behind such a claim could be studied in class tutorials, and issues such as study design, participant selection, sample size, statistical and clinical significance could be discussed. Research evidence used as basis for guidelines for a specific treatment recommendation could be used as study-material, requiring students to consider results of a meta-analysis of randomised controlled trials of effectiveness, with a tutor-led discussion featuring concepts such as effect size, confidence intervals, sampling error, heterogeneity and publication bias. When on placement in primary care, the GP could discuss with the students how they would explain the risks and benefits (expressed in relative and absolute terms) associated with, for example, starting or continuing hormone replacement therapy, or choosing a contraceptive method, using information typically available to the clinician.

• Use examples from actual published research in relevant medical journals covering topics in which students are likely to be interested in learning. For instance, learning about p-values could be embedded within consideration of an article reporting the results of a clinical trial of treatment regimes for Type I Diabetes.

• Integrate as much as possible with other subjects so statistics teaching does not stand-alone, separate from other elements of the curriculum.

• Introduce the subject early in undergraduate training and reinforce through-out the course, concentrating mainly on interpretation rather than carrying out statistical methods.

• Provide opportunities, closely linked to the point of need, for more in-depth and tailored guidance and training for those students undertaking student research projects requiring them to conduct analysis.

• Use a variety of teaching methods, to suit both student learning style and appropriateness of the method for specific content. Ensure that some of the teaching is practical and interactive. For instance, seminars for discussion, lectures for overviews or introductions to a topic, online materials for directed self-study or self-assessment, role-play for risk communication, computer-based workshops for specific data-handling skills and classroom practicals for random sampling exercises.

• Ensure teaching is set at a level appropriate for students, taking account of the diverse educational backgrounds of current undergraduates. Optional sessions for students requiring remedial or more advanced training may be required to ensure that the needs of all students are met.

## Conclusions

In this study a large sample of practising doctors made suggestions on why, what and how statistics should be taught to medical undergraduates. The main themes to emerge were making the teaching explicitly relevant to future practice, and comments on the content of teaching, and teaching methods and organisation. Integration with, and application to, clinical learning and use of real and/or current research as examples are recommendations to work on. Using interactive teaching methods, where statistics and research methods are incorporated with other subjects wherever possible and appropriate, and building on and reinforcing statistical learning throughout the undergraduate course seems likely to maintain learning and to confirm its relevance. Even with recommendations such as those provided here, developing statistical teaching to best meet the needs of future doctors is unlikely to be easy in a crowded curriculum with large cohorts of students and widening ability. Success is most likely to be achieved by closer co-operation between teachers of statistics and clinical teachers identifying learning opportunities for statistics within the clinical curriculum rather than devising more and more complex stand-alone statistics courses. Improvements in teaching statistics to medical students should improve the understanding of statistical concepts and reduce the incidence of misconceptions among clinicians and medical researchers.

## Competing interests

The authors declare that they have no competing interests.

## Authors' contributions

SM, GMP, LSw, LSh & SL contributed to the conception and design of the study, SM, GMP, & LSw contributed to data collection, analysis and interpretation, SM, GMP & LSw contributed to drafting and to critical revision of the manuscript. LSh & SJL contributed to critical revision of the paper. All authors approved the final version of the manuscript.

## Pre-publication history

The pre-publication history for this paper can be accessed here:

http://www.biomedcentral.com/1472-6920/10/75/prepub
